# A Case of Acute Leukemia Following Remission of COVID-19 Infection; an Urge to Search for a Probable Association 

**Published:** 2021-07-24

**Authors:** Seyyed Mojtaba Nekooghadam, Afshin Moradi, Kimia Karimi Toudeshki, Mehdi Pishgahi

**Affiliations:** 1 Internal Department, Shohada-e-Tajrish Hospital, Shahid Beheshti University of Medical Sciences, Tehran, Iran.; 2 Pathology Department, School of Medicine, Shahid Beheshti University of Medical Sciences, Tehran, Iran.; 3 Cardiology Department, Shohada-e-Tajrish Hospital, Shahid Beheshti University of Medical Sciences, Tehran, Iran.

**Keywords:** Leukemia, COVID-19, Association, Case Reports

## Abstract

Many aspects of Covid-19 infection, especially its complications and long-term health consequences are still unknown. Several reports concerning concomitant covid-19 infection and hematological disorders have been published recently. We aim to present a unique case of acute myeloid leukemia in a 61-year-old man with a previous history of covid-19 infection 40 days ago, with near complete resolution of signs and symptoms attributable to covid-19 infection. After 3 weeks, the patient presented again with progressive weakness, nausea, vomiting, and epigastric pain. No evidence of active covid-19 infection was observed. Instead, remarkable drop of hemoglobin and platelets relative to the values recorded in the discharge notes of the patient was observed. Further workup of the patient revealed evidence of acute leukemia and severe bone marrow involvement. We decided to present the current case as a concerning probable association.

## 1. Introduction:

Coronavirus disease 2019 (covid-19), which was first detected in Wuhan, China on December 2019, rapidly became a global threat and currently has affected almost all countries in the world. World Health Organization (WHO) declared Covid-19 as a pandemic on March 11, 2020 (1). Although hematological changes including lymphopenia, leukocytosis, neutrophilia, and thrombocytopenia have been reported widely, concomitant covid-19 infection and hematological malignancies is not so common (2). Here, we aim to present a unique case of acute myeloid leukemia in a 61-year-old man with a previous history of covid-19 infection 40 days ago, with near complete resolution of signs and symptoms attributable to covid-19 infection.

## 2. Case Presentation:

A 61-year-old man presented with complaints of weakness, nausea, vomiting, and epigastric pain. His past medical history was just relevant for a recent covid-19 infection about 40 days ago presenting with symptoms of myalgia, cough, shortness of breath and weakness. The admission course of the patient was complicated by bilateral pneumothorax and pneumomediastinum, which had been treated via bilateral thoracostomy. After the resolution of mediastinal and pleural complications and significant resolution of initial signs and symptoms, the patient was discharged home in good general condition and in his usual state of health. Final laboratory workups at the day of discharge from the hospital included white blood cell (WBC) count of 11.6×10^3^/μL (4–9×10^3^/μL), hemoglobin level of 13.2g/dl (12.5-16g/dl) and platelet count of 153×10^3^/μL (140-450×10^3^/μL). Also, electrolyte panels, renal function tests, and venous blood gas analysis were all within the normal limits. Chest computed tomography (CT) scan performed on the last day of his hospital stay, was unremarkable; except for minimal mediastinal air and bilateral reticular opacities in favor of resolving phase of covid-19 infection. 

About 40 days later, the patient again presented to the emergency department of our hospital complaining of a new progressive disabling weakness, accompanied by anorexia, epigastric pain, nausea, vomiting and non-bloody watery diarrhea. The patient also reported an unintentional weight loss of about 17 kg over the past 40 days. He persistently declined any episode of fever, cough, chest pain, abnormal bleeding, ecchymosis, petechiae, purpura or urinary symptoms.

Initial vital signs were notable only for sinus tachycardia and mild tachypnea with a rate of 25cycles/minute. Body temperature, blood pressure, and oxygen saturation were all within normal limits. Close physical examination revealed a conscious, moderately ill, and fatigued patient with significant emaciation. Conjunctival pallor, dry mucosa and grade 1 symmetric non-pitting limb edema were also detected. Lungs and heart were clear to auscultation. Abdominal examination was unremarkable. 

Complementary laboratory workups, including a polymerase chain reaction (PCR) for SARSCoV-2 by nasopharyngeal swab, were requested. Returning results were found to be surprisingly abnormal revealing a dramatic fall in hemoglobin and platelet count compared to values recorded on the discharge notes of the patient 40 days ago. Covid-19 PCR was reported negative. Electrocardiography was also unremarkable. Laboratory test results on the current admission are summarized in table 1. 

Concerned by the history of low molecular weight heparin use, recommended at the time of his previous discharge, and in accordance with our previous experiences regarding occurrence of retroperitoneal hematoma in patients treated with anticoagulants, a contrasted lung and abdominopelvic CT scan was requested for the patient. New imaging studies were irrelevant except for some parenchymal changes compatible with resolved past covid-19 infection, no hematoma was observed in the retroperitoneal, pelvic, and rectus sheet. Also, no evidence of intestinal ischemia, intramural bowel hematoma, and tumor lesion was observed.

**Figure 1:**
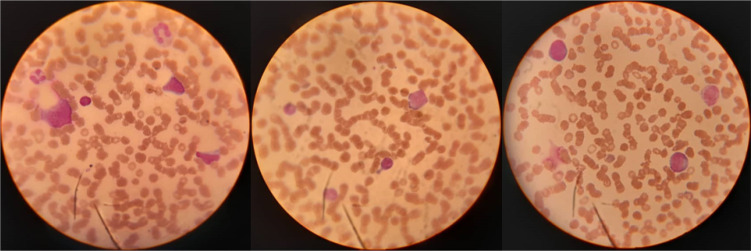
Peripheral blood films show numerous blast-like cells with fine chromatin and high nucleus-cytoplasm (N/C) ratio along with remarkable rouleaux formation and thrombocytopenia (Wright Giemsa stain, 100×).

**Figure 2:**
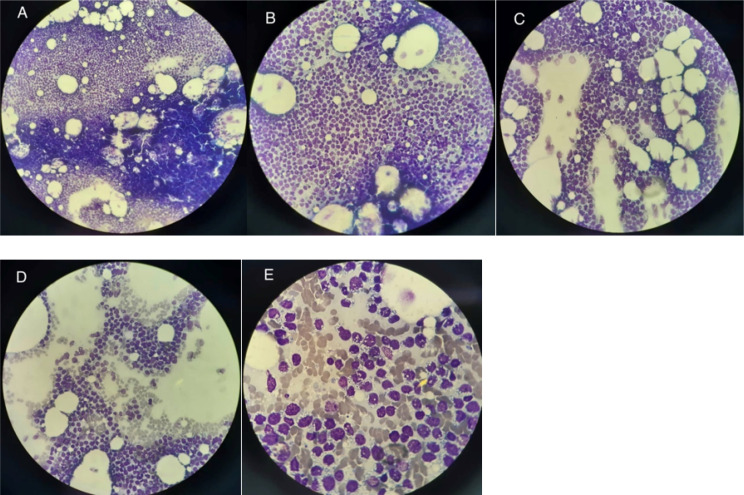
Bone marrow aspiration specimen revealed large blasts with open chromatin and one to three nucleoli with vacuolated cytoplasm constitute more than 20% of nucleated marrow cells (Wright Giemsa stain, (A) 10×, (B, C, D) 40×, (E) 100×)

**Figure 3:**
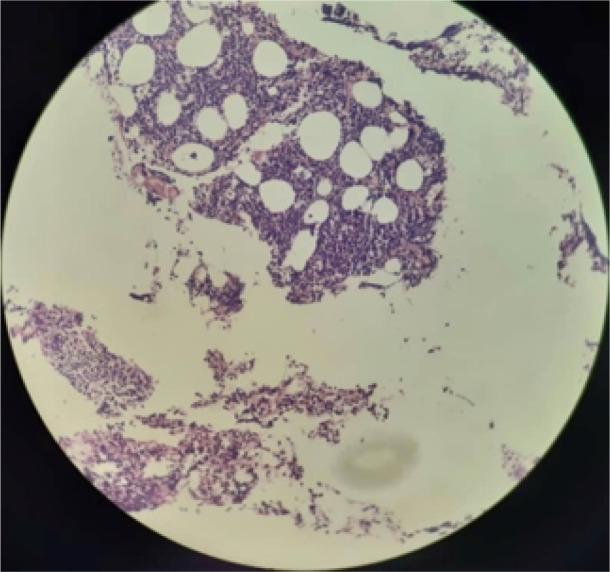
Bone marrow biopsy revealed hypercellular marrow for age mostly replaced by a diffuse infiltration of blasts (Hematoxylin Eosin stain, 10×)

Despite initial improvement of his symptoms, severe fatigue, lethargy, and somnolence supervened. Peripheral blood smear was requested, which revealed strange abnormalities including premature myeloid series, severe thrombocytopenia, and some abnormal large unfamiliar cells resembling blasts in a myelodysplastic/myeloproliferative picture (Figure 1). Subsequently, bone marrow biopsy and aspiration from the left posterior superior iliac spine of the patient was done immediately. Aspiration smears surprisingly revealed sheets of large vacuolated blasts encompassing more than 20% of nucleated marrow cells (Figure 2). The cells were morphologically reported to be compatible with acute leukemia of lymphoid linage (ALL L3) or myeloid linage (AML M4) by pathologists. Bone marrow biopsy showed hypercellular marrow for age mostly replaced by a diffuse infiltration of blasts, decrease in megakaryocytes, plasma cells less than 5%, and lymphocytes less than 10% of nucleated bone marrow cells (Figure 3). The patient received platelet transfusions and was finally admitted to the hematology service for more workups and to start chemotherapy.

## 3. Discussion:

Covid-19 has many different pathologic aspects. Hematological changes in covid-19 infection are mostly lymphopenia, leukocytosis, neutrophilia, and thrombocytopenia (3). Qiubai Li et al. studied on hematological features of 1449 hospitalized patients with covid-19 disease in five hospitals in Wuhan and in total, the median amounts of hemoglobin level was 132 g/L in maximum and 119 g/L in minimum (normal range: 115-150), median amounts of white blood cell count was 7×10^9^/L in maximum and 5×10^9^/L in minimum (normal range: 3,5-10) and for platelets, median amounts was 258×10^9^/L in maximum and 176×10^9^/L in minimum (normal range: 125-350). In this study, in patients who finally died, the median amount for minimum level was 105 g/L for hemoglobin, 6×10^9^/L for white blood cell count and 80×10^9^/L for platelets. Also, four dynamic co-variates including platelets correlated with an increased risk of death (4). 

In patients with covid-19 infection, different mechanisms such as antiviral drugs, damage to lung, cellular immunity and cytokines, dysfunction of bone marrow microenvironment, decreased production of thrombopoietin (TPO), increased platelet clearance and increased platelet consumption may lead to thrombocytopenia (5). Also, patients may develop neutropenia or pancytopenia, but on very rare occasions (6). Furthermore, Samuel E. Weinberg et al. reported the presence of atypical lymphocytes with medium to large size, loosely condensed chromatin and moderate to deep basophilic cytoplasm in the peripheral blood smear of recently admitted patients with covid-19 infection in addition to lymphopenia or lower normal range of lymphocyte count. These atypical lymphocytes were also seen in bronchial alveolar lavage smears of some patients with the same morphology as those found in the blood smear. It appeared that the percentage of atypical lymphocytes in total lymphocytes does not correlate with the severity of the disease (7). Also, Tariq N. Aladily et al. reported a transient increase in blast count following covid-19 infection in a 54-year-old man. He presented with fever, fatigue, and petechial rash on lower limbs. Complete blood count test showed 3.9×10^9^/L for white blood cell count, 84×10^9^/L for platelet count and a hemoglobin concentration of 111g/L. Few circulating blasts, constituting 6% of WBCs were seen on the peripheral blood smear and bone marrow aspiration demonstrated hypocellular marrow, approximately 23% blasts and exuberant proliferation of monocytes and lymphocytes. The patient was conservatively treated for covid-19 infection and without specific therapy, the blood cells gradually increased until they were normalized within 2 weeks with the spontaneous disappearance of blast from peripheral blood and normal cellularity in the second bone marrow aspiration. Multicolor flow cytometry confirmed being negative for acute leukemia. In certain conditions a reversible increase in blast count mimicking acute leukemia can occur. In these rare conditions such as acute infection, antibiotic treatment or blood transfusion, an immune-mediated process, also called spontaneous remission of acute leukemia, may occur followed by disappearance of blasts. Although it is mentioned that most patients ultimately develop a relapse of acute leukemia, the patient reported in this study was free of acute leukemia for five weeks (8). But on the other hand, concomitant Chronic Lymphocytic Leukemia (CLL) with covid-19 infection has also been reported. The complete blood count of a 49-year-old man without any past medical history who presented with a moderate covid-19 infection with fever and mild shortness of breath, showed a high white blood cell count with absolute lymphocytosis and flow cytometry confirmed the diagnosis of CLL. In contrast to the lymphopenia, lymphocytosis was mentioned as an unusual finding in covid-19 infection and malignancy or other infections should be considered in these cases. The interaction between covid-19 infection and CLL is still not known and more studies are recommended (9). Furthermore in another study, a 41-year-old male with no significant past medical history and the presentation of fever, watery diarrhea and epistaxis, a positive PCR for SARS-CoV-2 and pancytopenia with >50% blasts on the peripheral blood smear was diagnosed with concurrent Acute Myeloid Leukemia with covid-19 infection, confirmed by bone marrow biopsy (10). In hematological patients with covid-19 infection, covid-19 manifestation during their routine treatments for hematological disease has been reported as atypical clinical features (including higher proportion of fatigue, expectoration and hemoptysis, related to severe anemia, thrombocytopenia and infection with other respiratory pathogens also atypical characteristics on CT imaging), defective viral clearance, and lower level of SARS-CoV-2-specific antibodies (11). But our patient had developed covid-19 40 days before, he was discharged in good condition and the complete blood count at the time of discharge had not revealed any remarkable abnormality. The patient presented 3 weeks after discharge, following remission of covid-19 infection, with new onset progressive weakness, severe thrombocytopenia, and normochromic microcytic anemia, and was finally diagnosed with severe acute leukemia. 

**Table1:** Laboratory findings of the patients in his current admission

**Variables**	**Result**	**Normal range**
White blood cells (/μL)	11.1×10^3^	4-9×10^3^
Absolute Lymphocyte count (/μL)	6.1×10^3^	1-4.8×10^3^
Absolute neutrophil count (/μL)	3.9×10^3^	2-6×10^3^
Absolute Eosinophil count (/μL)	0.3×10^3^	<0.5×10^3^
Absolute Monocyte count (/μL)	0.8×10^3^	0.2-0.95×10^3^
Hemoglobin (g/dl)	10.5	12.5-16
Mean corpuscular volume (FL)	75.73	78-98
Mean corpuscular hemoglobin (pg)	27.7	27-35
Platelet count (/μL)	26×10^3^	140-450×10^3^
C-reactive protein (mg/dl)	14.6	<10
Erythrocyte sedimentation rate (mm/hr)	18	0-10
Blood urea nitrogen (mg/dl)	20	5-25
Creatinine (mg/dl)	0.88	0.5-1.5
Prothrombin time (seconds)	14	11-14
International normalized ratio	1.1	0.83-1.14
D. dimer (ng/ml)	900	<500
Lactate dehydrogenase (U/L)	1764	220-500
Total iron-binding capacity (μg/dl)	210	250-450
Serum iron (mg/dl)	154	50-168
Ferritin (ng/ml)	>800	20-290
Sodium (meq/L)	134	135-150
Potassium (meq/L)	3.85	3.5-5
**Arterial blood gas analysis**		
pH	7.27	7.35-7.45
PCO2 (mmHg)	16.7	35-45
HCO3 (mmHg)	7.5	22-26

## 4. Conclusion:

It has not been more than about a year since the first covid-19 infection was reported and despite many researches, many aspects of the disease, especially its complications and long-term health consequences are still unknown. Seeing this patient, there is a great deal of concern as to whether there is an association between covid-19 infection or perhaps the drugs used to treat this disease and the risk of developing acute leukemia. If there really is such an association, it is a great warning and there may be a need to review current therapies used for covid-19 infection treatment. It appears that further studies and follow-up for people with a history of covid-19 infection is needed.

## 5. Declarations:

### 5.1 Ethical consideration

Informed consent was obtained for the publication of clinical data of the patient.

### 5.2 Conflicts of interest

None.

### Acknowledgment

None.

### 5.3 Authors' contributions

All authors meet the standard criteria of authorship contribution based on the recommendations of the International Committee of Medical Journal Editors.
